# The Impact of Risk Factor Control on Health-Related Quality of Life in Individuals with High Cardiovascular Disease Risk: A Cross-sectional Study Based on EQ-5D Utility Scores in Inner Mongolia, China

**DOI:** 10.1007/s44197-021-00028-y

**Published:** 2022-01-03

**Authors:** Ning Cao, Zhihui Hao, Liwei Niu, Nan Zhang, Hao Zhu, Han Bao, Tao Yan, Xin Fang, Xiaoqian Xu, Lehui Li, Yan Liu, Yuan Xia, Xiong Su, Xingguang Zhang

**Affiliations:** 1 Public Health College, Inner Mongolia Medical University, Hohhot, China; 2 People’s Hospital of Inner Mongolia Autonomous Region, Hohhot, China

**Keywords:** Cardiovascular disease, High-risk individuals, Health-related quality of life, EQ-5D, Risk factors

## Abstract

**Objectives:**

To assess the impact of cardiovascular disease (CVD) risk factor control on health-related quality of life (HRQoL), as well as the other influencing factors of HRQoL among high CVD risk individuals.

**Methods:**

From 2015 to 2017, residents of six villages or communities in Inner Mongolia, selected using a multi-stage stratified cluster random sampling method, were invited to complete a questionnaire and undergo physical examination and laboratory testing. We selected participants whose predicted 10-year risk for CVD exceeded 10% as those with high CVD risk. HRQoL in individuals with high CVD risk was investigated based on the EuroQol-5 Dimension (EQ-5D) scale. The Chinese utility value integral system was used to calculate EQ-5D utility scores, and the Tobit regression model was used to analyze the influencing factors of HRQoL among individuals with high CVD risk.

**Results:**

Of 13,359 participants with high CVD risk, 65.63% reported no problems in any of the five dimensions; the most frequently reported difficulty was pain/discomfort. The median utility score was 1.000 (0.869, 1.000). Participants with hypertension, and uncontrolled glycemic and blood lipids had lower HRQoL. In addition, sex, age, living environment, education level, household income, and medical insurance were influencing factors of HRQoL.

**Conclusion:**

Sex, age, living environment, education level, household income, medical insurance, hypertension, and whether glycemic and blood lipids control or not are related to HRQoL of high CVD risk individuals.

## Background

Cardiovascular disease (CVD), including ischemic heart disease, stroke, heart failure, peripheral arterial disease, and a variety of other cardiac and vascular disorders, is the major cause of morbidity and mortality globally. From 1990 to 2019, the number of people suffering from CVD is nearly doubled. In addition, the number of CVD fatalities has grown by roughly 50% during the last several decades [1]. CVD remains the leading cause of disease burden worldwide, and China has the highest burden of CVD [2]. In China, stroke and ischemic heart disease were the main causes of mortality and disability-adjusted life years (DALYs) in 2017 [3].

Inner Mongolia is a multi-ethnic region in northern China. Because of the unique geographical and climatic qualities, as well as the well-developed animal husbandry, high-salt, high-fat diets, and high rate of drinking alcohol are common among local residents. Furthermore, the burden of CVD illness in Inner Mongolia is quite high, owing mostly to ischemic stroke and ischemic heart disease [3, 4]. As a result, CVD is the primary health issue at the moment in Inner Mongolia.

CVD is characterized by a high disability rate and a long course of disease. Therefore, health-related quality of life (HRQoL) has gained increased attention as a critical indicator in the evaluation of CVD [5]. HRQoL is a multifactorial concept that includes physical and mental health, symptoms, functional status, and overall health perception [6]. Studies have revealed that CVD is significantly associated with impaired HRQoL [6–9].

Some studies have focused on HRQoL among individuals with a high CVD risk. A high-risk individual for CVD is defined as predicted 10-year risk for CVD exceeding 10%. Multiple CVD risk factors (such as smoking, obesity, hypertension, diabetes, and dyslipidemia) are present in high-risk individuals, or these risk factors are present at a high degree [10]. A study in eight European countries showed that patients at risk of CVD had impaired HRQoL [5]. A South Korean study found that a 10-year CVD risk exceeding 20% is an independent predictor of impaired HRQoL [11]. All of these evidences revealed that the high risk of CVD being associated with impaired HRQoL.

Risk factor control in high CVD risk individuals is seen as an essential method of CVD primary prevention. However, several studies have demonstrated that the control of CVD risk factors was insufficient [12–15]. The same may be said for our studies on high CVD risk individuals in Inner Mongolia [16]. However, a few studies have been conducted to determine if uncontrolled risk factors impact HRQoL in high CVD risk patients. In this study, we focused on a population at high risk of CVD in Inner Mongolia, with the goal of assessing the impact of CVD risk factor control on HRQoL, as well as the other influencing factors of HRQoL among high CVD risk individuals, to serve as a model for enhancing the HRQoL of high CVD risk individuals, promoting primary CVD prevention, and lowering the disease burden of CVD in Inner Mongolia.

## Materials and Methods

### Study Participants

From 2015 to 2017, permanent inhabitants in Inner Mongolia were sampled using a multi-stage stratified cluster sampling method. We chose six counties (districts) based on geographic areas, Helingeer County of Hohhot City, Haibowan District of Wuhai City, Zhungeer Banner of Erdos City, Aohan Banner of Chifeng City, Horqin Rightwing Front Banner of Xingan League, and Manzhouli City of Hulunbeier City were the selected counties (districts) in Inner Mongolia. Then, we selected one village (community) from each of the six counties (districts) listed above. Permanent inhabitants aged 35–75 years from six different villages or communities took part in the survey. Detailed sampling methods have been described previously [16]. Finally, we include a total of 70,380 permanent inhabitants of Inner Mongolia.

### Data Collection

Selected residents were invited to complete a questionnaire and to undergo a physical examination and laboratory testing. The questionnaire included socio-demographic characteristics (sex, age, ethnicity, annual household income, educational level, marital status, residence area, and medical insurance), lifestyle, and disease history. Physical examination and laboratory testing included measurement of blood pressure, blood glucose, lipid levels, height, and weight. Measure the investigator's height and weight, while he or she is dressed comfortably and without shoes. For blood pressure measurement, the electronic sphygmomanometer (Omron HEM-7430) was utilized to measure blood pressure and measure blood pressure in a static, emotionally stable condition, took the average blood pressure after testing two times as the final value. The participants were invited for measurement of blood glucose and blood lipids at least 10 h after fasting the day before the examination. Blood glucose was measured by a glucose analyzer (BeneCheck PD-G001-2, Taiwan, China). The blood lipid test was measured by a rapid lipid analyzer (CardioChek PA Analyzer; Polymer Technology Systems, Indianapolis, Indiana, USA). The participants took height and weight measurements while wearing light clothing and without shoes.

### Assessment of High CVD Risk

We conducted the assessment according to the “Atherosclerotic cardiovascular disease risk assessment procedure” in the Chinese Guidelines for Prevention of Cardiovascular Disease (2017) [10]; a 10-year CVD risk ≥ 10% was determined as the high-risk individual for CVD. The findings of questionnaires, physical examinations, and laboratory testing were used to assess the CVD risk of permanent residents. Moreover, extremely high-risk individuals with a medical history of coronary heart disease, myocardial infarction, or stroke had been eliminated from this study, and the results had a greater reference importance for primary CVD prevention. Following the inclusion of participants based on the aforementioned criteria and the removal of missing value, a total of 13,359 high CVD risk participants were included in the study.

### Measurement of Health-Related Quality of Life (HRQoL)

Participants with a high CVD risk were assessed using questionnaires designed to evaluate their cardiovascular status in detail, including physical activity, dietary information, and other lifestyle factors, family history, menstrual history, and health-related quality of life (HRQoL). We used the EuroQol-5 Dimension (EQ-5D) scale to evaluate HRQoL. This scale is an essential tool to assess HRQoL with the benefits of simplicity, ease of use, and good reliability. The EQ-5D-3L scale contains five dimensions (mobility, self-care, usual activities, pain/discomfort, and anxiety/depression); each dimension has three levels of response or severity: no problems, some/moderate problems, and severe problems [6, 11].

For this study, the EQ-5D utility scores were calculated based on the Chinese utility value integral system [17]. If all five dimensions are at the first level, this indicates a state of complete health, and the utility score is 1. If all five dimensions are at the third level, this indicates the worst state of health, and the utility score is − 0.149. Therefore, the range of EQ-5D scores is − 0.149 to 1.000.

### Definition of Covariates

This study focuses on the influence of known risk factors control on HRQoL in the high CVD risk group. These risk factors for CVD include smoking, obesity, hypertension, diabetes, and dyslipidemia. Smoking was defined as consuming at least 1 stick of tobacco every day for more than 6 months. An ex-smoker is someone who used to smoke, but had not smoked in more than 6 months. Body mass index (BMI) was calculated as weight in kilograms divided by height (in meters) squared. A BMI of less than 18.5 kg/m^2^ was regarded thinnish, a BMI of 18.5–24.0 kg/m^2^ was considered normal, a BMI of 24.0–27.9 kg/m^2^ was considered overweight, and a BMI of at least 28.0 kg/m^2^ was deemed obese [18]. Hypertension was defined as a systolic blood pressure (SBP) of 140 mm Hg or higher, a diastolic blood pressure (DBP) of 90 mm Hg or higher, or self-reported use of antihypertensive medications in the previous 2 weeks [19]. Diabetes was defined as fasting blood glucose levels of 126 mg/dL or above, or self-reported diabetes, or administration of a hypoglycemic medication or insulin injections in the previous 2 weeks. Dyslipidemia has been characterized as a total cholesterol (TC) of 240 mg/dL or higher, or a low-density lipoprotein cholesterol (LDL-C) of 160 mg/dL or higher, or a high density lipoprotein cholesterol (HDL-C) of 40 mg/dL or lower, or triglyceride level (TG) of 200 mg/dL or higher, or use of lipid-lowering medicine in the previous two weeks [20].

Ex-smokers were regarded as smoking-controlled, while participants with a BMI < 28 were considered as weight-controlled. Control of hypertension was defined as an average SBP < 140 mm Hg and an average DBP < 90 mm Hg among participants with hypertension. Control of diabetes was defined as a fasting blood glucose < 126 mg/dL among participants with diabetes. Control of dyslipidemia was defined as a TC < 240 mg/dL, an LDL-C < 160 mg/dL, an HDL-C ≧ 40 mg/dL, and a TG < 200 mg/dL among participants with dyslipidemia.

Current smokers were regarded as smoking uncontrolled, and obesity was considered as weight uncontrolled. Uncontrolled hypertension, diabetes, and dyslipidemia were characterized as high CVD risk persons who did not achieve the treatment goal. The assigned values of each variable are shown in Table 1.

**Table 1:** Variable assignments

Variables	Assignments
Sex	Male = 0; female = 1
Age	35–44 = 0; 45–54 = 1; 55–64 = 2; 65–75 = 3
Education level	Primary school = 0; middle or high school = 1; college = 2
Living environment	Urban = 0; rural = 1
Marital status	Single = 0; married = 1
Household income (¥/year)	≤ 50,000 (¥/year) = 0; > 50,000 (¥/year) = 1
Medical insurance	No = 0; yes = 1
Drinking status	No drinking = 0; drinking^a^ = 1
Smoking status	Non-smoker = 0; ex-smoker = 1; current smoker = 2
BMI	Normal and thinnish = 0; overweight = 1; obesity = 2
Hypertension	No = 0; hypertension and controlled = 1; hypertension but uncontrolled = 2
Diabetes	No = 0; diabetes and controlled = 1; diabetes but uncontrolled = 2
Dyslipidemia	No = 0; dyslipidemia and controlled = 1; dyslipidemia but uncontrolled = 2

*BMI* body mass index

^a^Drinking was defined as consuming alcohol at least once per month over the previous 12 months

### Statistical Analysis

Descriptive statistics were used to summarize the utility scores of EQ-5D. As the utility score distribution was skewed and censored at 1, the median and 25th and 75th percentiles [*M* (*P*_25_, *P*_75_)] were used. Kruskal–Wallis tests or Wilcoxon rank-sum tests were used to detect the differences in EQ-5D scores among the various subgroups. The Tobit regression model was used to assess the influencing factors of EQ-5D utility scores. Statistical analyses were performed using IBM SPSS version 25.0 (IBM Corp., Armonk, NY, USA) and Stata version 15.0 (Stata Corp LLC, College Station, TX, USA). Two-sided *P* < 0.05 was considered statistically significant.

## Results

### Characteristics of Individuals with High CVD Risk

We included 13,359 participants in Inner Mongolia with a high risk of CVD. The mean age of participants was 56.57 ± 8.94 years, with 5885 men (mean age 55.75 ± 9.43 years) and 7474 women (mean age 57.22 ± 8.48 years). One quarter of the population at high CVD risk was current smokers, while 44.1 and 34.1% were overweight and obese. The prevalence of hypertension was 91.9%, with only a tiny fraction of participants had their blood pressure under control. The prevalence of diabetes and dyslipidemia was 27.1 and 53.4%, respectively; blood glucose and lipid control rates were similarly poor. Demographic and clinical characteristics of the 13,359 participants are presented in Table 2.

**Table 2:** Demographic characteristics of individuals with high CVD risk in Inner Mongolia [*n* (%)]

Factors	Groups	Men	Women	Total
Ethnic group	Han	5308 (90.2)	6819 (91.1)	12,118 (90.7)
	Mongol	563 (9.6)	648 (8.7)	1211 (9.1)
	Other^a^	14 (0.2)	16 (0.2)	30 (0.2)
Living environment	Rural	4116 (69.9)	5419 (72.5)	9535 (71.4)
	Urban	1769 (30.1)	2055 (27.5)	3824 (28.6)
Education level	Primary school	1759 (29.9)	4147 (55.5)	5906 (44.2)
	Middle or high school	3149 (53.5)	2823 (37.8)	5972 (44.7)
	College	977 (16.6)	504 (6.7)	1481 (11.1)
Marital status	Single	232 (3.9)	600 (8.0)	832 (6.2)
	Married	5653 (96.1)	6874 (92.0)	12,527 (93.8)
Household income	≤ 50,000 (¥/year)	4952 (84.1)	6774 (90.6)	11,726 (87.8)
	> 50,000 (¥/year)	933 (15.9)	700 (9.4)	1633 (12.2)
Medical insurance	No	528 (9.0)	666 (8.9)	1194 (8.9)
	Yes	5357 (91.0)	6808 (91.1)	12,165 (91.1)
Drinking status	No	3151 (53.5)	7098 (95.0)	10,249 (76.7)
	Yes	2734 (46.5)	376 (5.0)	3110 (23.3)
Smoking status	Non-smoker	2233 (37.9)	6731 (91.7)	8904 (67.1)
	Ex-smoker	806 (13.7)	259 (3.5)	1065 (8.0)
	Current smoker	2846 (48.4)	6731 (6.5)	8964 (24.9)
BMI	Normal and thinnish	1233 (21.0)	1635 (22.0)	2868 (21.5)
	Overweight	2597 (44.2)	3274 (44.0)	5871 (44.1)
	Obesity	2047 (34.8)	2535 (34.1)	4582 (34.4)
Hypertension	No	581 (9.9)	508 (6.8)	1089 (8.2)
	Hypertension and controlled	91 (1.5)	54 (0.7)	145 (1.1)
	Hypertension but uncontrolled	5213 (88.6)	6912 (92.5)	12,125 (90.8)
Diabetes	No	4205 (71.5)	5529 (74.0)	9734 (72.9)
	Diabetes and controlled	80 (1.4)	83 (1.1)	163 (1.2)
	Diabetes but uncontrolled	1600 (27.2)	1862 (24.9)	3462 (25.9)
Dyslipidemia	No	2686 (45.6)	3545 (47.4)	6231 (46.6)
	Dyslipidemia and controlled	48 (0.8)	139 (1.9)	187 (1.4)
	Dyslipidemia but uncontrolled	3151 (53.5)	3790 (50.7)	6941 (52.0)

*CVD* cardiovascular disease, *BMI* body mass index

^a^The other ethnic groups including Manchu, Korean, Oroqen, Ewenki, and so on

### Distribution of Self-Reported Health States Among Individuals with High CVD Risk

Table 3 summarizes the percentage of self-reported problems (No, moderate, or severe problems) on the EQ-5D questionnaire. In total, 8768 individuals reported no problems in any of the five dimensions (65.63%). Across the five dimensions, problems in the pain/discomfort dimension were the most frequently reported (30.9%), followed by anxiety/depression (24.4%). Problems in the self-care dimension were the least reported (0.4%).

**Table 3:** Distribution of EQ-5D self-reported health states among individuals with high CVD risk [*n* (%)]

	No problem	Moderate problem	Extreme problem
Mobility	13,199 (98.8)	155 (1.2)	5 (0.0)
Self-care	13,298 (99.6)	54 (0.4)	6 (0.0)
Usual activities	13,199 (98.8)	155 (1.2)	5 (0.0)
Pain/discomfort	9233 (69.1)	4093 (30.7)	33 (0.2)
Anxiety/depression	10,104 (75.6)	3247 (24.3)	8 (0.1)

*CVD* cardiovascular disease

### EQ-5D Utility Scores Among Individuals with High CVD Risk

The EQ-5D utility score among individuals with high CVD risk in Inner Mongolia was 1.000 (0.869, 1.000), indicating that more than half of those with high CVD risk had no difficulties in any of the EQ-5D's five dimensions. EQ-5D utility scores among participants with high CVD risk, according to different characteristics, are shown in Table 4. We found statistically significant differences in utility scores by sex, age group, ethnic group, education level, living environment, household income, medical insurance status, and whether or not hypertension, diabetes, and dyslipidemia were controlled (all *P* < 0.05). There was no significant difference in utility scores by marital status, drinking, smoking, and different BMI groups (all *P* > 0.05).

**Table 4:** Comparison of EQ-5D utility scores among individuals with high CVD risk

Variables	Groups	*n*	The EQ-5D utility scores		*Z/χ*^2^	*P*
			$\overline{x }\pm s$	*M* (*P*_*25*_, *P*_*75*_)		
Gender	Male	5885	0.94 ± 0.094	1.000 (0.869, 1.000)	− 7.571	< 0.001
	Female	7474	0.93 ± 0.098	1.000 (0.869, 1.000)		
Age	35–44	1394	0.95 ± 0.089	1.000 (0.875, 1.000)	39.774	< 0.001
	45–54	4133	0.93 ± 0.095	1.000 (0.869, 1.000)		
	55–64	5116	0.93 ± 0.097	1.000 (0.869, 1.000)		
	65–75	2716	0.93 ± 0.099	1.000 (0.869, 1.000)		
Ethnic group	Han	12,118	0.93 ± 0.096	1.000 (0.869, 1.000)	7.166	0.028
	Mongol	1211	0.94 ± 0.097	1.000 (0.869, 1.000)		
	Other^a^	30	0.94 ± 0.091	1.000 (0.874, 1.000)		
Living environment	Rural	9535	0.94 ± 0.092	1.000 (0.869, 1.000)	13.491	< 0.001
	Urban	3824	0.92 ± 0.104	1.000 (0.783, 1.000)		
Education level	Primary school	5906	0.93 ± 0.099	1.000 (0.869, 1.000)	14.997	0.001
	Middle or high school	5972	0.94 ± 0.093	1.000 (0.869, 1.000)		
	College	1481	0.94 ± 0.094	1.000 (0.869, 1.000)		
Marital status	Single	832	0.93 ± 0.097	1.000 (0.869, 1.000)	− 1.535	0.125
	Married	12,527	0.93 ± 0.096	1.000 (0.869, 1.000)		
Household income	≤ 50,000 (¥/year)	11,726	0.93 ± 0.097	1.000 (0.869, 1.000)	− 4.200	< 0.001
	> 50,000 (¥/year)	1633	0.94 ± 0.091	1.000 (0.869, 1.000)		
Medical insurance	No	1194	0.93 ± 0.100	1.000 (0.783, 1.000)	− 2.223	0.026
	Yes	12,165	0.94 ± 0.096	1.000 (0.869, 1.000)		
Drinking status	No	10,249	0.94 ± 0.096	1.000 (0.869, 1.000)	− 1.326	0.185
	Yes	3110	0.93 ± 0.096	1.000 (0.869, 1.000)		
Smoking status	Non-smoker	8964	0.93 ± 0.096	1.000 (0.869, 1.000)	2.256	0.324
	ex-smoker	1065	0.93 ± 0.096	1.000 (0.869, 1.000)		
	Current Smoker	3330	0.94 ± 0.096	1.000 (0.869, 1.000)		
BMI	Normal and thinnish	2874	0.93 ± 0.097	1.000 (0.869, 1.000)	0.893	0.640
	Overweight	5871	0.93 ± 0.097	1.000 (0.869, 1.000)		
	Obesity	4614	0.94 ± 0.095	1.000 (0.869, 1.000)		
Hypertension	No	1089	0.92 ± 0.100	1.000 (0.869, 1.000)	42.635	< 0.001
	Hypertension and controlled	145	0.91 ± 0.110	1.000 (0.783, 1.000)		
	Hypertension but uncontrolled	12,125	0.94 ± 0.095	1.000 (0.869, 1.000)		
Diabetes	No	9734	0.94 ± 0.095	1.000 (0.869, 1.000)	7.553	0.023
	Diabetes and controlled	163	0.92 ± 0.106	1.000 (0.869, 1.000)		
	Diabetes but uncontrolled	3462	0.93 ± 0.098	1.000 (0.869, 1.000)		
Dyslipidemia	No	6231	0.94 ± 0.094	1.000 (0.869, 1.000)	32.084	< 0.001
	Dyslipidemia and controlled	187	0.93 ± 0.096	1.000 (0.869, 1.000)		
	Dyslipidemia but uncontrolled	6941	0.93 ± 0.098	1.000 (0.869, 1.000)		

*CVD* cardiovascular disease, BMI body mass index

^a^The other ethnic groups including Manchu, Korean, Oroqen, Ewenki, and so on

### Influencing Factors of HRQoL in Individuals with High CVD Risk

Regression coefficients obtained in the Tobit regression model are shown in Table 5. Except for ethnic groups, all other factors were influencing factors of HRQoL among individuals with high CVD risk. The EQ-5D utility scores of women were lower than those of men (*P* < 0.001). Older people had lower scores than younger people (All *P* values are < 0.001). Individuals living in rural areas had higher scores than those living in urban areas (*P*  <0.001). Individuals with higher education levels had significantly higher utility scores (*P* were < 0.001 and 0.026, respectively). Individuals with higher medical insurance and high household income had significantly higher utility scores (All *P* values are 0.001).

**Table 5:** Influencing factors of health-related quality of life in patients with high CVD risk

Factors	Coef	95% CI	SE	*t*	*P*
Gender (ref. = male)					
Female	− 0.009	(− 0.0125, − 0.0057)	0.0017	− 5.30	< 0.001
Age (ref. = 35–44)					
45–54	− 0.0122	(− 0.0180, − 0.0063)	0.0030	− 4.10	< 0.001
55–64	− 0.0154	(− 0.0212, − 0.0096)	0.0030	− 5.21	< 0.001
65–75	− 0.0127	(− 0.0191, − 0.0062)	0.0033	− 3.88	< 0.001
Ethnic group (ref. = Han)					
Mongol	0.0012	(− 0.0045, 0.0069)	0.0029	0.42	0.673
Other^a^	0.0117	(− 0.0222, 0.0456)	0.0173	0.68	0.499
Living environment (ref. = rural)					
Urban	− 0.0327	(− 0.0367, 0.0288)	0.0020	− 16.45	< 0.001
Education level (ref. = primary school)					
Middle or high school	0.0124	(0.0086, 0.0161)	0.0019	6.41	< 0.001
College	0.0072	(0.0009, 0.0136)	0.0032	2.54	0.026
Household income (ref. = ≤ 50,000 ¥/year)					
> 50,000 (¥/year)	0.0144	(0.0089, 0.0199)	0.0027	5.15	< 0.001
Medical insurance (ref. = uninsured)					
Insured	0.0142	(0.0083, 0.0200)	0.0030	4.75	< 0.001
Hypertension (ref. = no)					
Hypertension and controlled	− 0.0155	(− 0.0217, − 0.0092)	0.0032	− 4.86	< 0.001
Hypertension but uncontrolled	− 0.0221	(− 0.0378, 0.0065)	0.0080	− 2.78	0.005
Diabetes (ref. = no)					
Diabetes and controlled	− 0.0063	(− 0.0210, 0.0083)	0.0075	− 0.85	0.397
Diabetes but uncontrolled	− 0.0043	(− 0.0080, − 0.0006)	0.0019	− 2.30	0.022
Dyslipidemia (ref. = no)					
Dyslipidemia and controlled	− 0.0059	(− 0.0197, 0.0079)	0.0070	− 0.84	0.403
Dyslipidemia but uncontrolled	− 0.0044	(− 0.0078, − 0.0009)	0.0017	− 2.50	0.013

*CVD* cardiovascular disease

^a^The other ethnic groups including Manchu, Korean, Oroqen, Ewenki, and so on

The findings revealed that patients with hypertension had lower health utility values than non-hypertensive patients, regardless of whether their blood pressure was controlled at a normal level (*P* were < 0.001 and 0.005, respectively). Patients with diabetes whose blood glucose was not controlled at the normal level had a poorer health utility value than non-patients (*P* = 0.022), while patients whose glucose was controlled at the level had a health utility value that was not statistically different from non-patients (*P* = 0.397). The same is true for those who had dyslipidemia. Patients whose blood lipids were not controlled at the normal level had a poorer health utility value than non-patients (*P* = 0.013), whereas patients whose lipids were controlled at the level had the same health utility value as non-patients (*P* = 0.403).

## Discussion

In the prevention and treatment of CVD, the HRQoL of individuals with high CVD risk has not attracted sufficient attention, despite the high risk of CVD being associated with impaired HRQoL in the previous studies. In the present large-scale study in Inner Mongolia, we evaluated patients’ HRQoL using the EQ-5D scale. Moreover, we identified that individuals with hypertension had lower HRQoL, regardless of whether their blood pressure was normal or not. Patients with diabetes or dyslipidemia who did not have effective blood glucose or lipid management had low HRQoL, as well. In addition, we discovered the following influencing factors on HRQoL in individuals with high CVD risk: female sex, older age, living in urban areas, lower education level, and lower household income. These findings are critical in the primary prevention of CVD.

The common scales used for HRQoL study are the EQ-5D scale and the short-form 36 health survey questionnaire (SF-36). The EQ-5D scale has a clear structure and is easy to operate, while the SF-36 questionnaire has too many question items, making the survey more time-consuming and laborious [21]. Additionally, EQ-5D is more suitable for people with poor health, while the sample investigated in this study is a high-risk population of CVD, so the health status is not very good, and the EQ-5D scale was proved friendlier to with lower education levels [22]. Due to the ceiling effect of the EQ-5D scale, the EQ-5D utility score was a censored variable, and the Tobit regression model used in this study is suitable for the study of censored data analysis.

We found that 65.63% of individuals with high CVD risk reported no problems in any of the five dimensions of the EQ-5D, which was better than the results of an HRQoL survey among Chinese and Australian patients with CVD [23–25]. Despite the fact that high CVD risk individuals have numerous risk factors, they have not acquired CVD. The HRQoL of the high-risk population is still better than that of CVD patients. Pain/discomfort was the most commonly reported problem, followed by anxiety/depression, which is consistent with results of 2008 and 2013 national health services surveys [26]. This reveals that harm to the physical and mental health of individuals with a high risk of CVD occurs to different degrees. Our finding also suggests that psychological problems should not be neglected in these patients, and we should strengthen the intervention of those by providing timely counseling to reduce their psychological burden [27].

According to Tobit regression model analysis, older age and female sex were associated with worse HRQoL, which is consistent with previous research among individuals with a high risk of CVD in other regions [5, 6]. One reason for this finding is likely that physiological functioning of the organism declines with increased age. Our finding also emphasizes the importance of strengthening self-health management education and health management services for older individuals. People living in rural regions obtained higher HRQoL than those living in urban areas, despite the fact that urban areas are typically thought to have better medical care conditions. However, urban areas may attract more patients with higher CVD risk, resulting in lower HRQoL of urban inhabitants. Furthermore, because the participants in this study were permanent residents, the results of this study would not be muddled by those who had lived in metropolitan regions for brief periods of time for medical treatment; hence, our results are relatively reliable. Individuals with a high risk of CVD who had higher levels of education and household income had higher HRQoL, which is consistent with previous research among individuals with a high risk of CVD in other regions and patients with established CVD in China [5, 6, 23, 28]. Patients with a greater degree of education have a completer and more correct grasp of physical health and may master more health-related knowledge than those with a lower level of education. Patients with higher incomes may have better access to health care. From another viewpoint, there is an unbalanced distribution of health status among individuals with high CVD risk. Attention is needed in populations with lower income and education levels. In this study, those with medical insurance had greater HRQoL; it helps to reduce the economic pressures of high CVD risk individuals. This demonstrates the significance of strengthening medical security.

According to our findings, whether or not the risk factors of high CVD risk individuals were properly controlled is an influencing factor of HRQoL. First, the findings reveal that risk factors, such as hypertension, dyslipidemia, obesity, and diabetes, were prevalent among high CVD risk individuals in Inner Mongolia. However, as demonstrated by our past study, risk factor control was not optimistic [16]. Previous studies have revealed that hypertension individuals' HRQoL is poorer than that of healthy people [22, 29–31]. Our study also found that patients with hypertension in the high CVD risk population had poorer HRQoL than non-hypertensive patients, regardless of whether their blood pressure is adequately controlled. Low HRQoL in patients with controlled blood pressure might be attributed to more intensive treatment to control blood pressure levels and the awareness of disease [32, 33]. Even if the result demonstrated that HRQoL was still lower in patients than in non-patients, we cannot deny that lowering blood pressure had a good effect, and even if the blood pressure was not under control, the HRQoL was still low. Uncontrolled blood pressure might aggravate some clinical symptoms and were thus lower HRQoL, and previous research has revealed that patients with controlled blood pressure had a greater HRQoL than individuals without control [33, 34].

Previous research revealed that the HRQoL of patients with diabetes was poorer than that of healthy people [35, 36]. However, our research proved that patients with diabetes whose blood glucose was not controlled at the normal level had a poorer HRQoL than non-patients, while there was no difference in HRQoL between patients with controlled blood glucose and non-patients, which demonstrated the significance of glycemic control in improving HRQoL. Previous research has shown that better glycemic control improves HRQoL, while greater HRQoL improves glycemic control. It indicates that in diabetes individuals, HRQoL and glycemic management have a bidirectional link [34, 37]. Similarly, blood lipid control is required for high CVD risk individuals. Our study revealed that for those who had dyslipidemia, patients whose blood lipids were not controlled at the normal level had a poorer HRQoL than non-patients, whereas there was no difference in HRQoL between patients with controlled blood lipids and non-patients. All of these suggest that improving medication adherence in high CVD risk individuals is critical, and medication nonadherence was considered as an unrecognized cardiovascular risk factor [5, 38]. Therefore, to enhance the HRQoL of the high CVD risk individuals, patients' medication adherence must be improved.

Our study had several limitations. First, as this study was conducted using cross-sectional data, it was not possible to assess the causal relationship between HRQoL and its influencing factors. Second, there was a ceiling effect in the EQ-5D-3L scale used in this study. As also reported in other studies, EQ-5D can also be less susceptible to describing mild-severe health levels.

## Conclusion

Among individuals with a high CVD risk in Inner Mongolia, pain/discomfort was the most commonly reported problem. In Inner Mongolia, HRQoL of high CVD risk individuals was related to socio-demographic variables such as sex, age, living environment, education level, household income, and medical insurance. Furthermore, risk factor control is also related to HRQoL. Individuals with hypertension, regardless of whether their blood pressure was normal or not, had lower HRQoL. Patients with diabetes or dyslipidemia who did not have effective blood glucose or lipid management had low HRQoL, as well. Intervention strategies to improve hypoglycemia and hypolipidemic medication adherence can improve the HRQoL of individuals at high CVD risk.

## Data Availability

Not applicable.

## Abbreviations

CVD: Cardiovascular disease
HRQoL: Health-related quality of life
EQ-5D: EuroQol-5 dimension scale
EQ-5D-3L: EuroQoL-5 dimension scale with 3 levels
BMI: Body mass index
SBP: Systolic blood pressure
DBP: Diastolic blood pressure
TC: Total cholesterol
HDL-C: High-density lipoprotein cholesterol
LDL-C: Low-density lipoprotein cholesterol
TG: Triglyceride
CI: Confidence interval
SE: Standard error
SF-36: The short-form 36 health survey questionnaire

## References

[1] Roth GA, Mensah GA, Johnson CO, Addolorato G, Ammirati E, Baddour LM (2020). Global burden of cardiovascular diseases and risk factors, 1990–2019: update from the GBD 2019 study. J Am Coll Cardiol.

[2] Zhao D, Liu J, Wang M, Zhang X, Zhou M (2019). Epidemiology of cardiovascular disease in China: current features and implications. Nat Rev Cardiol.

[3] Zhou M, Wang H, Zeng X, Yin P, Zhu J, Chen W (2019). Mortality, morbidity, and risk factors in China and its provinces, 1990–2017: a systematic analysis for the Global Burden of Disease Study 2017. Lancet.

[4] Liu S, Li Y, Zeng X, Wang H, Yin P, Wang L (2019). Burden of Cardiovascular Diseases in China, 1990–2016: findings From the 2016 Global Burden of Disease Study. JAMA Cardiol.

[5] Thiem U, Ludt S, Wensing M, Szecsenyi J, van Lieshout J, Rochon J (2011). Predictors of health-related quality of life in patients at risk for cardiovascular disease in european primary care. PLoS ONE..

[6] Petek D, Petek-Ster M, Tusek-Bunc K (2018). Health behavior and health-related quality of life in patients with a high risk of cardiovascular disease. Slov J Public Health.

[7] Dyer MTD, Goldsmith KA, Sharples LS, Buxton MJ (2010). A review of health utilities using the EQ-5D in studies of cardiovascular disease. Health Qual Life Outcomes.

[8] Tran BX, Moir MP, Thai TPT, Nguyen LH, Ha GH, Nguyen THT (2018). Socioeconomic inequalities in health-related quality of life among patients with cardiovascular diseases in Vietnam. Biomed Res Int.

[9] De Smedt D, Kotseva K, De Backer G, Wood D, Van Wilder L, De Bacquer D (2020). EQ-5D in coronary patients: what are they suffering from? Results from the ESC EORP European Survey of Cardiovascular Disease Prevention and Diabetes (EUROASPIRE IV) Registry. Qual Life Res.

[10] Chinese Guidelines for Prevention of Cardiovascular Diseases (2017) Editorial Committee of Chinese Journal of Cardiovascular Diseases. Guidelines for prevention of cardiovascular disease in china. Chin J Cardiol. 2018;46:10–25. 10.3760/cma.j.issn.0253-3758.2018.01.004 29374933

[11] Ko H-Y, Lee J-K, Shin J-Y, Jo E (2015). Health-related quality of life and cardiovascular disease risk in Korean adults. Korean J Fam Med..

[12] Lu J, Lu Y, Yang H, Bilige W, Li Y, Schulz W (2019). Characteristics of high cardiovascular risk in 1.7 million Chinese adults. Ann Intern Med..

[13] Lu J, Lu Y, Wang X, Li X, Linderman GC, Wu C (2017). Prevalence, awareness, treatment, and control of hypertension in China: data from 1·7 million adults in a population-based screening study (China PEACE Million Persons Project). Lancet.

[14] Cai L, Li X, Cui W, You D, Golden AR (2018). Trends in diabetes and pre-diabetes prevalence and diabetes awareness, treatment and control across socioeconomic gradients in rural southwest China. J Public Health (Oxf).

[15] Pan L, Yang Z, Wu Y, Yin R-X, Liao Y, Wang J (2016). The prevalence, awareness, treatment and control of dyslipidemia among adults in China. Atherosclerosis.

[16] Xi Y, Cao N, Niu L, Zhu H, Bao H, Qiao L (2021). Prevalence and treatment of high cardiovascular disease risk in Inner Mongolia, China. Rev Cardiovasc Med.

[17] Liu GG, Wu H, Li M, Gao C, Luo N (2014). Chinese time trade-off values for EQ-5D health states. Value Health.

[18] Bureau of Disease Prevention and Control, Ministry of Health, People's Republic of China (2012). Nutrition improvement work management method. J Nutr.

[19] Chinese Hypertension Prevention and Treatment Guidelines Revision Committee, Hypertension Alliance (China), Cardiology Branch of Chinese Medical Association, Hypertension Professional Committee of Chinese Medical Doctor Association, Hypertension Branch of China Association for the Promotion of International Healthcare Exchanges, Hypertension Branch of Chinese Geriatric Society (2018). Guidelines for the prevention and treatment of hypertension in China. Chin J Cardiovasc Med.

[20] Chinese Joint Committee on the Revision of Guidelines for Prevention and Treatment of Adult Dyslipidemia (2016). Chinese guidelines on prevention and treatment of dyslipidemia in adults. Chin J Circ..

[21] Liang M, Fu X, Gao P, Zhu W (2014). Comparative analysis on EuroQol-5 dimensions and ShortForm6D in quality of life scale. Chin Health Econ..

[22] Wang C, Lang J, Xuan L, Li X, Zhang L (2017). The effect of health literacy and self-management efficacy on the health-related quality of life of hypertensive patients in a western rural area of China: a cross-sectional study. Int J Equity Health.

[23] Deng Q, Liu W (2020). Multilevel analysis of health related quality of life of patients with cardiovascular disease and its determinants. J Shandong Univ (Health Sci)..

[24] Mei Y-X, Wu H, Zhang H-Y, Hou J, Zhang Z-X, Liao W (2021). Health-related quality of life and its related factors in coronary heart disease patients: results from the Henan Rural Cohort study. Sci Rep.

[25] McCaffrey N, Kaambwa B, Currow DC, Ratcliffe J (2016). Health-related quality of life measured using the EQ-5D-5L: South Australian population norms. Health Qual Life Outcomes.

[26] Yao Q, Liu C, Zhang Y, Xu L (2019). Changes in health-related quality of life of Chinese populations measured by the EQ-5D-3 L: a comparison of the 2008 and 2013 National Health Services Surveys. Health Qual Life Outcomes.

[27] Yang D, Tang S (2018). Health-related quality of life and its influencing factors in patients with chronic diseases in Sichuan, Hebei and Gansu based on Tobit model. Chin Gen Pract.

[28] Wang X, Wang L, Fan K, Qu H, Zhang J, YanPing R (2020). Health-related quality of life and its influencing factors in stroke patients in rural areas of Western China: a survey based on EQ-5D scale. Chin J Mult Organ Dis Elder.

[29] Liang Z, Zhang T, Lin T, Liu L, Wang B, Fu AZ (2019). Health-related quality of life among rural men and women with hypertension: assessment by the EQ-5D-5L in Jiangsu, China. Qual Life Res.

[30] Wong ELY, Xu RH, Cheung AWL (2019). Health-related quality of life among patients with hypertension: population-based survey using EQ-5D-5L in Hong Kong SAR, China. BMJ Open..

[31] Zhang Y, Zhou Z, Gao J, Wang D, Zhang Q, Zhou Z (2016). Health-related quality of life and its influencing factors for patients with hypertension: evidence from the urban and rural areas of Shaanxi Province, China. BMC Health Serv Res..

[32] Trevisol DJ, Moreira LB, Fuchs FD, Fuchs SC (2012). Health-related quality of life is worse in individuals with hypertension under drug treatment: results of population-based study. J Hum Hypertens.

[33] Yan R, Gu H-Q, Wang W, Ma L, Li W (2019). Health-related quality of life in blood pressure control and blood lipid-lowering therapies: results from the CHIEF randomized controlled trial. Hypertens Res.

[34] Lee CJ, Park WJ, Suh J-W, Choi E-K, Jeon DW, Lim S-W (2020). Relationship between health-related quality of life and blood pressure control in patients with uncontrolled hypertension. J Clin Hypertens (Greenwich).

[35] Alshayban D, Joseph R (2020). Health-related quality of life among patients with type 2 diabetes mellitus in Eastern Province, Saudi Arabia: a cross-sectional study. PLoS ONE.

[36] Zhuang Y, Ma Q-H, Pan C-W, Lu J (2020). Health-related quality of life in older Chinese patients with diabetes. PLoS ONE.

[37] Testa MA, Simonson DC (1998). Health economic benefits and quality of life during improved glycemic control in patients with type 2 diabetes mellitus: a randomized, controlled, double-blind trial. JAMA.

[38] Munger MA, Van Tassell BW, LaFleur J (2007). Medication nonadherence: an unrecognized cardiovascular risk factor. MedGenMed.

